# Carfilzomib triggers cardiotoxicity by suppressing SENP1-mediated deSUMOylation of DDX17: SENP1 alleviates Cfz cardiotoxicity

**DOI:** 10.3724/abbs.2025121

**Published:** 2025-10-09

**Authors:** Sheng Wang, Jingjing Wang, Xin Li, Zhigao Dai, Tiantian Li, Yixuan Wang, Ziyi Peng, Mengqi Wang, Hao Cheng, Linchuang Jia, Danchen Su, Mu Qiao, Jingya Wang, Ying Xie, Jing Guo, Xiaozhi Liu, Tong Liu

**Affiliations:** 1 Department of Pathophysiology School of Basic Medical Science Tianjin Medical University Tianjin 300070 China; 2 Shandong Provincial Key Laboratory of Precision Oncology Shandong Cancer Hospital and Institute Shandong First Medical University and Shandong Academy of Medical Sciences Jinan 250117 China; 3 Houston Methodist Cancer Center Weill Cornell Medicine Houston TX 77030 USA; 4 Department of Cardiology Hebei Port Group Qinhuangdao Hospital of Integrated Traditional Chinese and Western Medicine Qinhuangdao 066000 China; 5 Tianjin Key Laboratory of Epigenetics for Organ Development of Preterm Infants Tianjin Fifth Central Hospital Tianjin 300450 China; 6 High Altitude Characteristic Medical Research Institute Huangnan Tibetan Autonomous Prefecture People’s Hospital Huangnan Prefecture 811399 China; 7 Tianjin Key Laboratory of Ionic-Molecular Function of Cardiovascular Disease Department of Cardiology Tianjin Institute of Cardiology Second Hospital of Tianjin Medical University Tianjin 300211 China

**Keywords:** cardio-oncology, carfilzomib, SENP1, DDX17, SUMOylation

## Abstract

Carfilzomib (Cfz) is a second-generation proteasome inhibitor approved for the treatment of relapsed/refractory multiple myeloma (RRMM). Previous studies have shown that Cfz is associated with a higher incidence of severe adverse cardiac effects than bortezomib (Btz); however, the underlying mechanisms remain to be elucidated. The aim of this study is to identify key regulators of cardiotoxicity induced by Cfz and to investigate the mechanisms by which these factors exert their effects. We establish a mouse model of cardiac toxicity induced by Cfz and confirm the phenotype through cardiac functional analysis, morphology assessment, myocardial fibrosis, and apoptosis analysis. We subsequently perform RNA sequencing to identify differentially expressed genes (DEGs) and further validate their functions and mechanisms. We find that Cfz induces myocardial hypertrophy and myocardial injury, along with the suppression of SENP1 expression in mouse heart tissues and *in vitro* cultured neonatal rat cardiomyocytes. Suppression of SENP1 exacerbates Cfz-induced injury and remodeling in cardiomyocytes by directly binding to and deconjugating the SUMO1-mediated SUMOylation of the RNA helicase DDX17. This process leads to a reduction in K-48 ubiquitin-linked polyubiquitination and degradation of DDX17, resulting in increased expressions of anti-apoptotic genes and maintenance of mitochondrial homeostasis. Therefore, the overexpression of SENP1 using AAV vectors alleviates Cfz-induced cardiotoxicity in mice. In summary, our findings reveal a previously unknown role of the SENP1-DDX17 axis in protecting against cardiotoxicity induced by Cfz, providing a potential foundation for developing therapeutic strategies to mitigate cardiac side effects in the clinical management of MM patients.

## Introduction

Multiple myeloma (MM) is the second most common hematological malignancy and is characterized by abnormal proliferation of plasma cells in the bone marrow niche and the presence of a monoclonal immunoglobulin protein [1]. Bortezomib (Btz) is the first proteasome inhibitor (PI) approved for the treatment of newly diagnosed MM (NDMM), and carfilzomib (Cfz) is the first second-generation PI approved for the management of refractory or relapsed MM (RRMM), both of which have substantially improved the clinical outcome of MM patients [2]. However, PIs inhibit proteasome activity in cardiomyocytes and are associated with a significant incidence of cardiotoxicity, including hypertension (HTN), ischemic heart disease (IHD), heart failure (HF), and ventricular arrhythmia (VA) [3]. Among the PIs approved for clinical application, carfilzomib is the most strongly associated with cardiotoxicity [4]. PIs trigger the transcriptional activation of signaling pathways, such as NF-κB and autophagy, and those involved in regulating nitric oxide homeostasis may contribute to these cardiotoxic effects [5]. The underlying molecular mechanisms of therapy-associated cardiotoxic effects have been increasingly elucidated, but detailed mechanistic illustrations are still lacking.

Protein posttranslational modifications (PTMs) are essential for regulating various physiological and pathological processes within cells [6]. SUMOylation, a reversible PTM, contributes to the pathogenesis of cardiovascular diseases [7]. The covalent binding of SUMO proteins to their target proteins is a dynamic and reversible process regulated by the coordinated activities of SUMO-conjugating and deconjugating enzymes. In mammalian cells, the most prevalent group of deconjugating enzymes is the sentrin-specific proteases (SENPs), which consists of six members: SENP1, SENP2, SENP3, SENP5, SENP6, and SENP7 [8]. Among these SENPs, SENP1 exerts a protective effect on cardiac dysfunction after myocardial injury by facilitating the proteasomal degradation of HSP90ab1 [9]. However, whether SENP1 is critical for the development of cancer and therapy-related cardiotoxicity, particularly in multiple myeloma and Cfz-based regimens, remains unknown.

In the present study, we developed *in vitro* and *in vivo* models of Cfz-induced cardiotoxicity and identified SENP1 as a key regulator of cardioprotection. We then explored the mechanism by which SENP1 regulates the stability of the DEAD-box helicase 17 (DDX17) protein and the apoptosis of cardiomyocytes and assessed the potential benefits of activating SENP1 in mitigating cardiotoxicity in mice.

## Materials and Methods

### Animal study and ethical approval

The animal study was approved by the Ethic Committee of Tianjin Medical University, and all the protocols conformed to the Ethical Guidelines of the World Medical Association Declaration of Helsinki and the Guide for the Care and Use of Laboratory Animals published by the US National Institutes of Health (publication No. 85-23, revised 1996). Wild-type C57BL/6 male mice, 8 weeks old, were obtained from SPF Beijing Biotechnology (Beijing, China). The mice were randomly divided into two groups (vehicle control, *n* = 10; Cfz treatment, *n* = 20) and fed with a standard rodent laboratory diet. After a two-week acclimatization period, the mice were intraperitoneally administered Cfz (8 mg/kg) or vehicle every two days for a total of eight administrations. The survival of the mice was monitored daily, and body weights were recorded every two days. On day 14, all the mice were anaesthetized for echocardiography and blood sample collection. The mice were subsequently euthanized by cervical dislocation, and heart samples were collected for further analysis.

### Echocardiography

The mice were anaesthetized with isoflurane (1.5%–2%), and their body temperature was maintained with a heating pad. Echocardiography was performed to evaluate the structure and function of the left ventricle using a Vevo2100 imaging system (Visual-Sonics Inc., Toronto, Canada). Two-dimensional images are recorded in parasternal long- and short-axis projections with guided M-mode and B-mode echocardiography at the midventricular level in both views. The data were averaged from three cardiac cycles. Averaged LV diastolic and systolic posterior wall thickness (LVPWd, LVPWs), LV diastolic and systolic internal dimensions (LVIDd, LVIDs) and interventricular septal thickness at diastole and systole (IVSd, IVSs) are measured. The fractional shortening percentage (% FS) was calculated as [(LVIDd–LVIDs)/LVIDd] × 100%, and the ejection fraction percentage (% EF) was calculated as [(LVIDd ^3^− LVIDs ^3^)/ LVIDd ^3^] × 100%. The strains are expressed as percentages.

### Histology

Hearts were harvested from euthanized mice, fixed overnight in 10% formalin, embedded in paraffin and serially sectioned at 5 μm. The sections were stained with hematoxylin and eosin (H&E) according to the manufacturer’s protocol for morphometric measurement. To observe collagen and fiber deposits, Masson’s trichrome and Sirius red staining were performed on the selected sections according to the manufacturer’s protocol. The sections were examined using PANNORAMIC™ Digital Slide Scanners (Pannoramic MIDI, Budapest, Hungary) and browsed via CaseViewer 2.4 software (3DHISTECH, Budapest, Hungary).

### Serology test

The levels of lactate dehydrogenase (LDH) activity, aspartate aminotransferase (AST) activity, creatine kinase-MB (CK-MB), alanine aminotransferase (ALT), albumin (ALB), blood urea nitrogen (BUN), and creatinine (Cr) in the serum were measured using the corresponding assay kits listed in the Supplementary Resources. All the experiments were performed according to the manufacturer’s protocol.

### Cell culture and *in vitro* treatment

H9C2 rat cardiomyocytes were obtained from the Cell Bank of Shanghai Institutes for Biological Sciences, Chinese Academy of Sciences (Shanghai, China). HEK293T cells were a kind gift from Prof. Yupeng Chen at the Department of Biochemistry and Molecular Biology, Tianjin Medical University. HEK293T and H9c2 cells were cultured in high-glucose DMEM supplemented with 10% fetal bovine serum (FBS), 100 U/mL penicillin, 100 mg/mL streptomycin, and 2 mM L-glutamine (Life Technologies, Carlsbad, USA). Neonatal rat ventricular myocytes (NRVMs) and cardiac fibroblasts were isolated from newborn rats within 3 days as previously described [10]. The primary cardiomyocytes were cultured *ex vivo* in DMEM supplemented with 15% FBS and 0.1 mM 5-bromo-2-deoxyuridine to inhibit the growth of nonmyocardial cells. These cells were cultured at 37°C in a humidified incubator with 5% CO _2_ and 95% air (v/v).

### RNA extraction and quantitative real-time PCR

Total RNA was extracted from heart tissues or cultured cells using Trizol reagent and converted to cDNA using the All-In-One 5× RT MasterMix (Abm, Vancouver, Canada). Quantitative real-time PCR (qPCR) was performed using EvaGreen 2× qPCR MasterMix (Abm) in the QuantStudio 3 Real-Time PCR System (Applied Biosystems, Foster City, USA). The primer pairs (synthesized by Tsingke Biotech, Shanghai, China) used for quantitative real-time PCR are listed in the Supplementary Resources.

The formula 2 ^–ΔΔCt^ was used to calculate the fold change in gene expression, and the average ΔCt value of at least three independent biological experiments was recorded. The expression level of each gene was normalized to that of *Gapdh*, which served as an endogenous internal control.

### Immunofluorescence and immunohistochemistry

For immunofluorescence analysis, the cells grown on confocal dishes were fixed in 4% paraformaldehyde (pH 7.4) for 10 min, and then the samples were treated with 0.5% (v/v) Triton X-100 for 15 min and blocked with 5% BSA for 30 min at 37°C. Then, the cell samples were incubated with an anti-ACTN2 antibody (A8939, ABclonal Technology, Wuhan, China), anti-TNNT2 antibody (A4914; ABclonal Technology), or anti-Vimentin antibody (A21648; ABclonal Technology) overnight at 4°C. After three washes with PBS, the samples were incubated with Alexa Fluor® 488 donkey anti-rabbit IgG (H + L) (R37718; Invitrogen, Carlsbad, USA) for 60 min at room temperature, and the nuclei were counterstained with DAPI. Images were acquired via an Olympus FV1000 IX81-SIM confocal microscope (Olympus, Tokyo, Japan). Images were merged using Adobe Photoshop CS6 (Adobe Systems, San Jose, USA). For the measurement of cardiomyocyte size, three adjacent sections were quantified using ImageJ (version 1.46r) software (National Institutes of Health, Bethesda, USA).

Standard immunohistochemistry was performed on mouse heart sections. Briefly, the sections were deparaffinized in xylene and rehydrated. After the samples were hydrated in gradient ethanol (from 100% to 70%), antigen retrieval was performed with protease K at 37°C for 15 min. H _2_O _2_ (3%) was used to block the activity of endogenous peroxidase. The sections were then incubated overnight at 4°C with anti-SENP1 (YT4236; Immunoway, San Jose, USA) or anti-DDX17 (A5731; ABclonal Technology) antibodies. After three washes in PBS, biotinylated secondary antibodies were added, and the samples were incubated for 1 h at room temperature, followed by color development with a DAB kit (K5361; Dako, Glostrup, Denmark). The sections were examined using PANNORAMIC™ Digital Slide Scanners and browsed by CaseViewer2.4 software.

### TdT-mediated dUTP nick-end labelling (TUNEL) staining

TUNEL staining of mouse heart sections was performed using the DeadEnd™ Fluorometric TUNEL System (G3250; Promega, Madison, USA) according to the manufacturer’s instructions. Briefly, deparaffinized and rehydrated tissue sections were incubated with 100 μL of proteinase K (20 μg/mL) for 8–10 min. After being washed and incubated with 100 μL of equilibration buffer for 5–10 min, the slides were incubated with 50 μL of rTdT incubation buffer at 37°C for 60 min in the dark. The reactions were terminated by incubation with 2× SSC for 15 min at room temperature. The slides were washed with PBS and then sealed with hard-set mounting medium supplemented with DAPI. The sections were subsequently examined using PANNORAMIC™ Digital Slide Scanners and browsed by CaseViewer2.4 software (3DHISTECH).

### Caspase 3/7 assay

The caspase 3/7 activity of cardiomyocytes was measured using the Caspase-Glo®3/7 Assay kit (G8091; Promega) and a Microplate Reader 550 (Bio-Rad Laboratories, Richmond, USA) in accordance with the manufacturer’s instructions.

### Cell viability assay

Cardiomyocyte viability was analyzed by the Cell Counting Kit-8 (CCK-8) assay and LDH release assay. For the CCK-8 assay, cardiomyocytes were seeded at 1 × 10 ^5^ cells/well in triplicate in 96-well plates and incubated at 37°C in 5% CO _2_. After incubation for 48 h, the CCK-8 reagent was added to each well and incubated for 2 h, after which the absorbance was read at 450 nm. The following formula was used to calculate cell viability: cell viability (%) = OD value of the treatment group/OD value of the control group × 100%. The IC _50_ of the cells was thus determined. For the LDH release assay, the level of LDH released into the cell culture medium was detected using an LDH cytotoxicity assay detection kit (A020-2; Jiancheng Bio, Nanjing, China) following the manufacturer’s instructions.

### RNA sequencing and analysis

RNA sequencing and analysis were performed using Cfz- and vehicle-treated NRVMs ( *n* = 2 per group) at 24 h. Total RNA was extracted using Trizol according to the manufacturer’s instructions. The quality of the purified RNA was detected on an Agilent 2100 Bioanalyzer (Agilent RNA 6000 Nano kit, Santa Clara, USA). cDNA libraries were amplified and sequenced using the DNBSEQ platform (BGI, Shenzhen, China). FastQC (version 0.11.8) was used to check the raw sequencing reads. Adapter trimming and low-quality filtering were performed via Cutadapt (v2.0) to obtain the clean reads. After the clean reads were obtained, HISAT was used to align the clean reads to the reference genome sequence, and Bowtie2 was used to align the clean reads to the reference gene sequence (Reference *Rattus norvegicus* Genome Version: GCF_000001895.5_Rnor_6.0 from NCBI). Gene expression was quantified by featureCounts (v1.6.0), and differential expressed genes (DEGs) between treatment groups were identified via DESeq2 (R version 3.3.2). A cut-off value of a fold change ≥ 1.5 and an adjusted *P* value ≤ 0.05 were used. All the DEGs were subjected to GSEA and GO and KEGG enrichment analyses.

### Protein extraction and western blot analysis

Cultured cells and tissue samples were lysed in RIPA buffer (50 mM Tris-HCl pH 7.5, 150 mM NaCl, 10 mM EDTA, 0.5% sodium deoxycholate, 1% NP-40, 1 mM sodium dodecyl sulfate, 10 μg/mL aprotinin, 1 mM phenylmethylsulfonyl fluoride, and 10 μg/mL leupeptin) supplemented with complete protease inhibitors (04693132001; Roche, Basel, Switzerland). Protein fractions were collected by centrifugation at 13,000 *g* at 4°C for 15 min. The protein concentration was determined via a BCA protein assay kit. Protein samples (50 μg) were separated via SDS-PAGE and transferred to nitrocellulose membranes (Pall Corporation, Washington, USA). The membranes were blocked with 5% nonfat milk for 1 h at room temperature and infiltrated overnight at 4°C with specific primary antibodies (all the antibodies used are listed in the Supplementary Resources). The membranes were washed three times in PBST, incubated with horseradish peroxidase-conjugated secondary antibodies for 1 h at room temperature, washed three times with PBST, and finally, the bands were visualized via the SuperSignal West Pico chemiluminescence substrate (34580; Thermo Fisher Scientific, Waltham, USA). The representative western blot images for at least three independent experiments are shown in the figures. GAPDH was used as a total cell protein loading control for normalization. The western blot band intensities were quantified via ImageJ software (version 1.46r).

### Co-immunoprecipitation (Co-IP)

For Co-IP assays, cells were harvested and lysed with NP-40 lysis buffer (50 mM Tris-HCl, pH 7.4, 150 mM NaCl) supplemented with complete protease inhibitors on ice for 30 min. The cell lysate was centrifuged for 20 min at 12,000 *g* at 4°C. For Co-IP of exogenously expressed proteins, the supernatant was incubated with anti-FLAG M2 affinity gel at 4°C overnight. For Co-IP of endogenously expressed proteins, the supernatant was incubated with the indicated primary antibody and protein A/G agarose beads at 4°C overnight. The immunocomplexes were washed four times with NP-40 lysis buffer, after which the pulled-down proteins were detected by western blot analysis as described above.

### Sliver staining and mass spectrometry

For identification of interacting proteins, the SDS-PAGE gel loaded with the total Co-IP samples. Sliver staining was performed using the Pierce™ Silver Stain kit (Pierce, Rockford, USA) according to manufacturer’s instructions. Briefly, the gel was washed twice in ultrapure water, fixed twice in 30% ethanol, and washed sequentially in 10% ethanol and ultrapure water. Then, the gel was sensitized with sensitizer working solution for 1 min and stained with stain working solution for 30 min after being washed with water. The gel was washed twice with ultrapure water for 20 s, developed with developer working solution for 2–3 min until bands appeared, and the reaction was stopped with 5% acetic acid for 10 min. The corresponding bands were removed, further excised and subjected to a mass spectrum assay.

### Transfection, virus packaging and infection

Transient transfections of HEK293T cells were performed using polyethyleneimine (PEI) in OPTI-MEM with a ratio of 1:4 to 1:6 of DNA:PEI. For viral production, HEK293T cells cultured in 10-cm dishes were transfected with 4 μg of pMD2. G and 6 μg of psPAX2 packaging plasmids, along with 8 μg of lentiviral expression vectors encoding target genes, as detailed in the Supplementary Resources. All primers used in this study were synthesized by BGI. The supernatant containing the viral particles was collected at 35 to 60 h posttransfection and subsequently concentrated 100-fold using polyethylene glycol (PEG) 8000 (Sigma-Aldrich, St Louis, USA). For viral infection, 1 × 10 ^6^ infected cells were seeded in 1 mL of fresh complete medium and cultured for 6 h, after which 50 μL of the concentrated viral supernatant and 8 μg/mL polybrene were added. The cells were subjected to centrifugation at 500 g for 45 min at 20°C. Following a 12-h incubation period postspin infection, the cells were cultured with fresh medium for an additional 48 h before further analysis. The efficiency of gene knockdown or overexpression was assessed by qPCR and western blot analysis.

### Statistical analysis

All the data were analyzed with GraphPad Prism 9.0 and are presented as the mean ± SEM, except otherwise. Differences between groups were determined using two-sided Student’s *t* test or one/two-way ANOVA with Tukey’s post hoc test. For Kaplan-Meier survival plots, significance was measured using the log-rank (Mantel-Cox) test. *P* values less than 0.05 were considered to indicate statistically significant differences. Other materials are provided in the Supplementary Resources.

## Results

### Carfilzomib induces cardiotoxicity *in vivo* and *in vitro*

**Figure 1:**
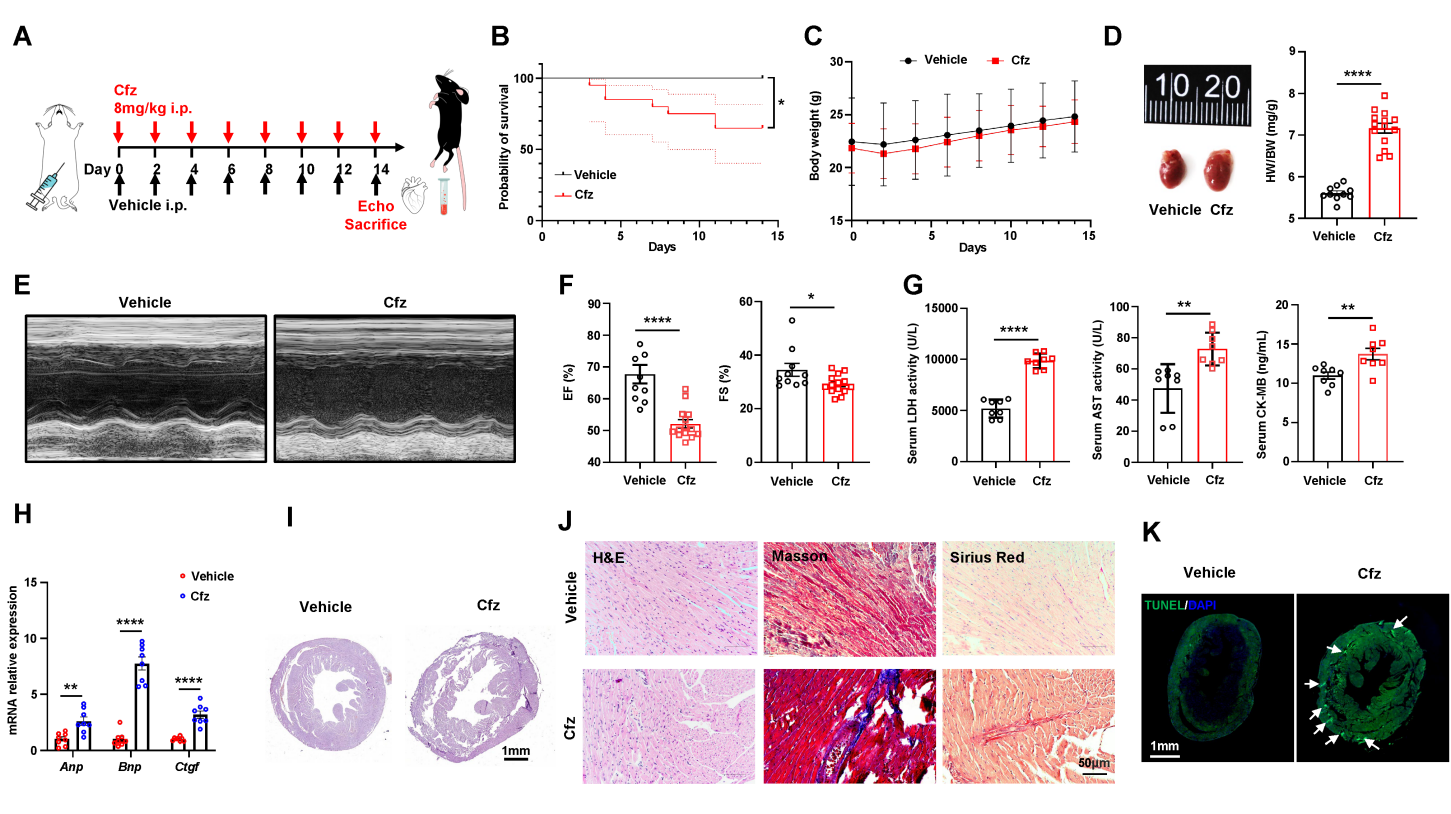
Figure 1 Carfilzomib induces cardiotoxicity *in vivo* (A) Experimental design and treatment schedule for carfilzomib-induced cardiotoxicity in adult male C57BL/6 mice. (Cfz group, n = 20; vehicle group, n = 10). (B) Survival curves of the mice in the two groups after two weeks of treatment (Cfz group: n = 20; vehicle group: n = 10). (C) Body weights of the two groups after two weeks of treatment (Cfz group: n = 14; vehicle group: n = 10). (D) Representative images of hearts showing the cardiac morphology of vehicle- and Cfz-treated mice (the minimum scale of the ruler is 1 mm), and heart weight-to-body weight (HW/BW) ratios were also analyzed after sacrificing and dissecting the two groups (Cfz group, n = 14; vehicle group, n = 10). (E) Representative M-mode echocardiography images of the vehicle and Cfz mice after two weeks of treatment. (F) Echocardiographic assessment of the ejection fraction (EF) and fractional shortening (FS) in vehicle-treated and Cfz-treated mice two weeks after treatment (Cfz group: n = 14; vehicle group: n = 10). (G) Mouse serum LDH, AST, and CK-MB levels in the two groups (Cfz group, n = 14; vehicle group, n = 10). (H) The mRNA levels of Anp, Bnp and Ctgf in the vehicle- and Cfz-treated left ventricles of mouse hearts were analyzed by RT-qPCR (n = 8). (I) Representative images of H&E-stained transverse heart sections from vehicle- and Cfz-treated mice (n = 4); scale bar, 1 mm. (J) Representative histological sections from hearts stained with H&E, Masson’s trichrome (MTT), and Sirius Red (n = 6); scale bar, 50 μm. (K) TUNEL staining of transverse heart sections from vehicle- and Cfz-treated mice (n = 4); scale bar, 1 mm; white arrows indicate apoptotic sites. Data are presented as the mean ± SEM and were analyzed by one-way ANOVA with Tukey’s multiple comparisons test (B–D and F–H). *P < 0.05, **P < 0.01, ***P < 0.001, ****P < 0.0001.

To determine the toxicity of Cfz to the heart, we administered Cfz to C57BL/6 mice for a period of 2 weeks ( Figure 1A). Cfz treatment resulted in mortality in the mice ( Figure 1B) without changing their overall body weight ( Figure 1C), and the heart value and weight were greater in the Cfz-treated group than in the vehicle control group ( Figure 1D). Notably, the parameters of left ventricular functions, including the ejection fraction (EF) ( Figure 1E), fractional shortening (FS) ( Figure 1F), left ventricular posterior wall thickness at end systole (LVPWs), left ventricular internal systolic diameter (LVIDs) and interventricular septal thickness at systole (IVSs) ( Supplementary Figure S1A–C), were significantly impaired in the Cfz treatment group, along with markedly elevated AST, LDH and CK-MB activity in the Cfz treatment group ( Figure 1G), and the expressions of the hypertrophic and fibrotic marker genes *Anp*, *Bnp* and *Ctgf* was increased ( Figure 1H). Changes in other cardiac indicators, including left ventricle posterior wall thickness diastole (LVPWd), left ventricular internal diameter end diastole (LVIDd), and interventricular septal thickness at diastole (IVSd), were not significantly altered ( Supplementary Figure S1D–F). In addition, to exclude off-target toxicity, we also examined the functions of the liver and kidney. Both liver and kidney function tests were performed by detecting the levels of alanine aminotransferase (ALT), albumin (ALB), blood urea nitrogen (BUN), and creatinine (Cr) ( Supplementary Figure S1G–J) in mouse serum, and the morphology results indicated that there was no significant difference in toxicity to other organs between the two groups ( Supplementary Figure S1K,L). Two weeks after administration, the Cfz-treated mice presented a larger cardiomyocyte cross-sectional area and thicker ventricular walls ( Figure 1I), increased cardiomyocyte size, exacerbated cardiac fibrosis ( Figure 1J), and increased apoptosis rates ( Figure 1K).

**Figure 2:**
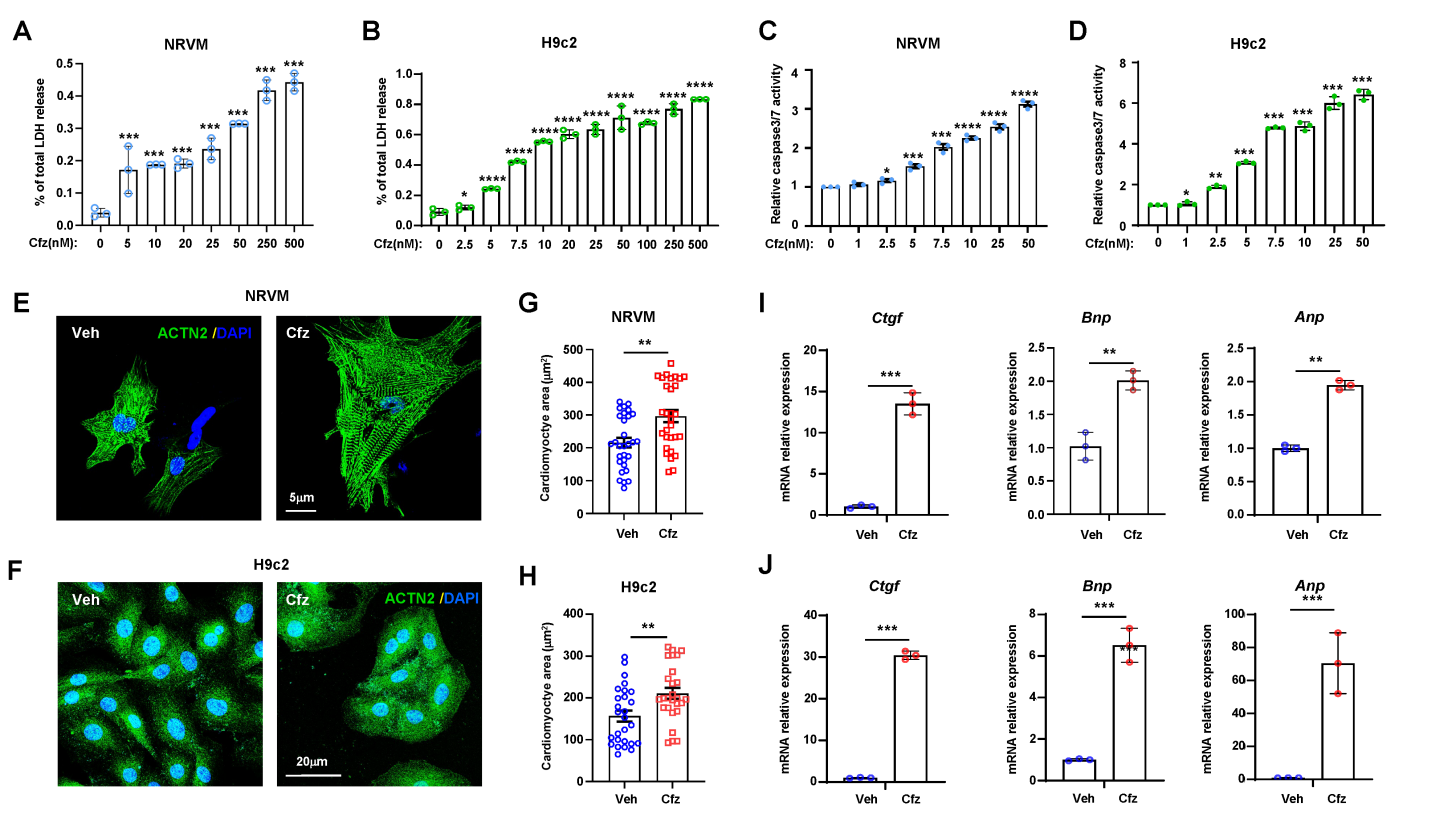
Figure 2 Carfilzomib causes myocardial cell injury and apoptosis *in vitro* (A,B) Percentage of total LDH released by LDH cytotoxicity assay in NRVMs or H9c2 cells pre-incubated with Cfz followed by gradient concentration for 24 h (n = 3). (C,D) Relative caspase 3/7 activity in NRVMs or H9c2 cells pre-incubated with Cfz for 24 h (n = 3). (E,F) Representative immunofluorescent confocal micrographs of the morphology of NRVMs (scale bar, 5 μm) or H9c2 cells (scale bar, 20 μm) stained with ACTN2 (green) and DAPI, which were treated with vehicle or 1 μM Cfz for 24 h. The data represent four independent experiments. (G,H) Quantitative analysis of the relative NRVM or H9c2 cell size by immunofluorescence staining. (I,J) mRNA levels of Ctgf, Bnp and Anp in vehicle- and Cfz-treated NRVMs or H9c2 cells for 24 h (n = 3). Data are presented as the mean ± SEM and were analyzed by two-way ANOVA (A–D) or Student’s t test (G–J). *P < 0.05, **P < 0.01, ***P < 0.001, ****P < 0.0001.

To further confirm the toxicity of Cfz in cardiomyocytes, we treated neonatal rat ventricular cardiomyocytes (NRVMs) and rat cardiomyocyte H9c2 cells with Cfz *in vitro*. The NRVMs were validated by the expression of alpha-actinin 2 (ACTN2), cardiac troponin T (TNNT2), and vimentin ( Supplementary Figure S2A), and Cfz had a lethal effect on NRVM and H9c2 viability ( Supplementary Figure S2B). A dose-dependent increase in LDH ( Figure 2A,B) and increased activity of caspase 3/7 in NRVMs and H9c2 cells were observed ( Figure 2C,D). Additionally, the toxic effect of Cfz was also reflected by the irregular alpha actinin and arrangement of larger cardiomyocytes in both NRVMs and H9c2 cells ( Figure 2E–H), along with the overexpressions of the hypertrophic-related genes *Ctgf*, *Bnp* and *Anp* ( Figure 2I,J). Collectively, these results indicate that Cfz induces myocardial hypertrophy, fibrosis, and apoptosis.

### SENP1 is downregulated by Cfz and is negatively correlated with the severity of cardiomyocyte damage

**Figure 3:**
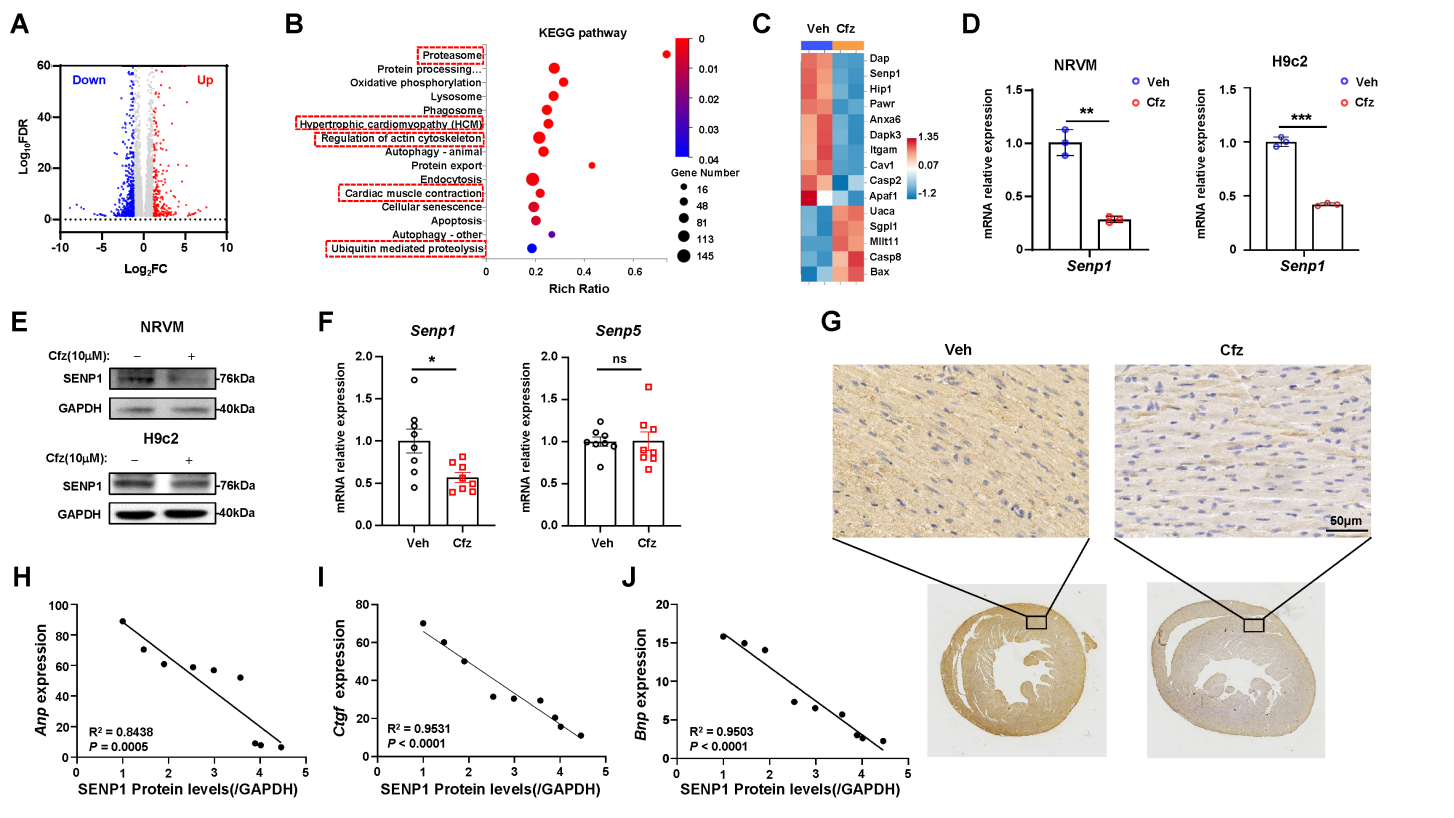
Figure 3 Cfz induces cardiotoxicity by suppressing SENP1 expression (A) Volcano plot of differentially expressed genes analyzed by bulk RNA sequencing in NRVM cells treated with vehicle or Cfz. Blue, downregulated genes; red, upregulated genes; gray, statistically nonsignificant genes. (B) KEGG pathway annotation analysis for DEGs with P < 0.05 via the DAVID method. (C) Heatmap of differentially expressed key genes of the apoptotic signaling pathway in NRVMs treated with Cfz or vehicle. (D) qPCR analysis of Senp1 expression in NRVM and H9c2 cells treated with 10 μM Cfz or vehicle for 24 h (n = 3/group). (E) Representative western blots of SENP1 protein levels in NRVM and H9c2 cells treated with 10 μM Cfz or vehicle for 24 h (n = 3/group). (F) qPCR analysis of Senp1 and Senp5 expressions in vehicle- and Cfz-treated left ventricles of mouse hearts (n = 8/group). (G) Representative immunohistochemical staining of SENP1 protein in the heart tissues of mice treated with vehicle or Cfz. Scale bar, 50 μm. (H-J) Pearson correlation coefficients between the protein level of SENP1 and the mRNA levels of Anp, Ctgf and Bnp in H9c2 cells treated with Cfz (n = 9). Data are presented as the mean ± SEM and were analyzed by Student’s t test (D and F) or linear regression analysis (H). *P < 0.05, **P < 0.01, ***P < 0.001.

To decipher how Cfz alters the transcription of cardiomyocytes, we employed bulk RNA sequencing of NRVM cells treated with vehicle or Cfz. We identified 6064 differentially expressed genes (DEGs) (adjusted *P* value < 0.05) ( Figure 3A), and KEGG pathway enrichment analysis revealed that the upregulated genes were related to the proteasome ( *Uchl1*, *NRIP3*), cardiomyopathy ( *Xirp2*, *Gdf15*), cytoskeleton ( *SPIRE2*, *DMTN*), and apoptosis ( *Tspan17*, *Sgpl1*), whereas the downregulated genes ( *Senp1*) were correlated with ubiquitin-mediated proteolysis ( Figure 3B,C). Consistently, Gene Ontology (GO) and GSEA analyses further revealed that DEGs were enriched in the ubiquitination, drug response, cardiac development, SUMOylation, proteasome, and ubiquitination degradation pathways in cardiomyocytes ( Supplementary Figure S3A–D).

Since Cfz irreversibly binds to the proteasome and affects ubiquitin-dependent proteasomal degradation [4], we speculated that the suppression of SENP1 may be an important candidate for treating cardiotoxicity. Indeed, Cfz treatment significantly suppressed the mRNA and protein levels of *SENP1*, both in NRVM cells and in heart tissues from Cfz-treated mice ( Figure 3D–F), but not in the homologue of SENP5 or other SENPs ( Supplementary Figure S4A,B). Significantly reduced SENP1 expression was observed in heart sections from mice ( Figure 3G), and SENP1 expression was negatively correlated with the expressions of cardiac injury markers in H9c2 cardiomyocytes ( Figure 3H–J).

### SENP1 deficiency exacerbates Cfz-induced cardiomyocyte toxicity

**Figure 4:**
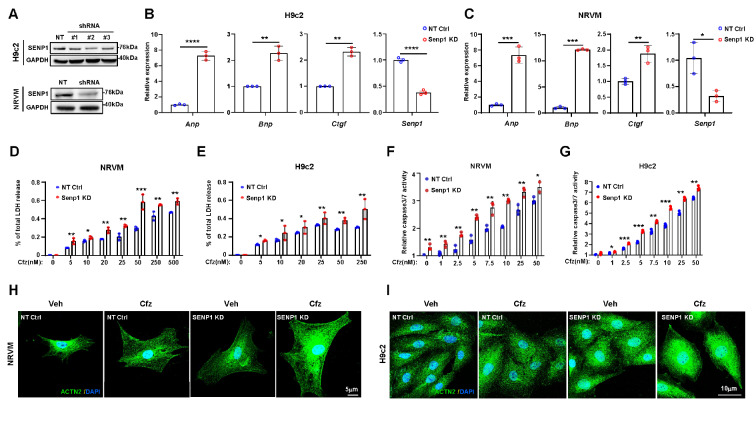
Figure 4 Knockdown of *SENP1* exacerbates myocyte injury (A) Representative western blots of SENP1 protein levels in H9c2 cells and NRVMs transfected with SENP1 shRNA or the corresponding nontargeting control for 48 h. (B,C) The mRNA levels of Senp1, Anp, Bnp and Ctgf were analyzed by RT-qPCR in SENP1-KD H9c2 or NRVM cells (n = 3). (D,E) Quantification of the percentage of total LDH release in NT Ctrl and SENP1-KD NRVM cells treated with Cfz for 24 h by LDH cytotoxicity assay and analysis of the difference between the two groups (n = 3). (F,G) Relative caspase 3/7 activity in NT Ctrl and SENP1-KD NRVM cells treated with Cfz for 24 h (n = 3). (H,I) Representative immunofluorescence confocal micrographs of the morphology of SENP1-KD NRVMs (scale bar, 5 μm) or H9c2 cells (scale bar, 10 μm) treated with vehicle or 10 μM Cfz for 24 h. Data are presented as the mean ± SEM and were analyzed by Student’s t test (B,C) or two-way ANOVA (D–G). *P < 0.05, **P < 0.01, ***P < 0.001, ****P < 0.0001.

To validate the role of SENP1 in the regulation of Cfz-induced cardiotoxicity, we measured the absence of SENP1 in cardiomyocytes by interfering with SENP1 expression in NRVMs and H9c2 cells ( Figure 4A). The expressions of hypertrophic markers and myocardial fibrosis-related genes were significantly augmented by *SENP1* deletion ( Figure 4B,C). In addition, cardiomyocyte damage was markedly accelerated, as evidenced by a dose-dependent increase in LDH release ( Figure 4D,E) and overactivation of Caspase3/7, which is a marker of apoptosis in SENP1-deficient cardiomyocytes ( Figure 4F,G). Moreover, *SENP1* knockdown increased the size of cardiomyocytes and disrupted the arrangement of myocardial fiber structures ( Figure 4H,I). Thus, these data confirm that the loss of SENP1 aggravates Cfz-induced cardiac dysfunction, apoptosis, and myocardial structural abnormalities.

### SENP1 interacts with and stabilizes the DDX17 protein

**Figure 5:**
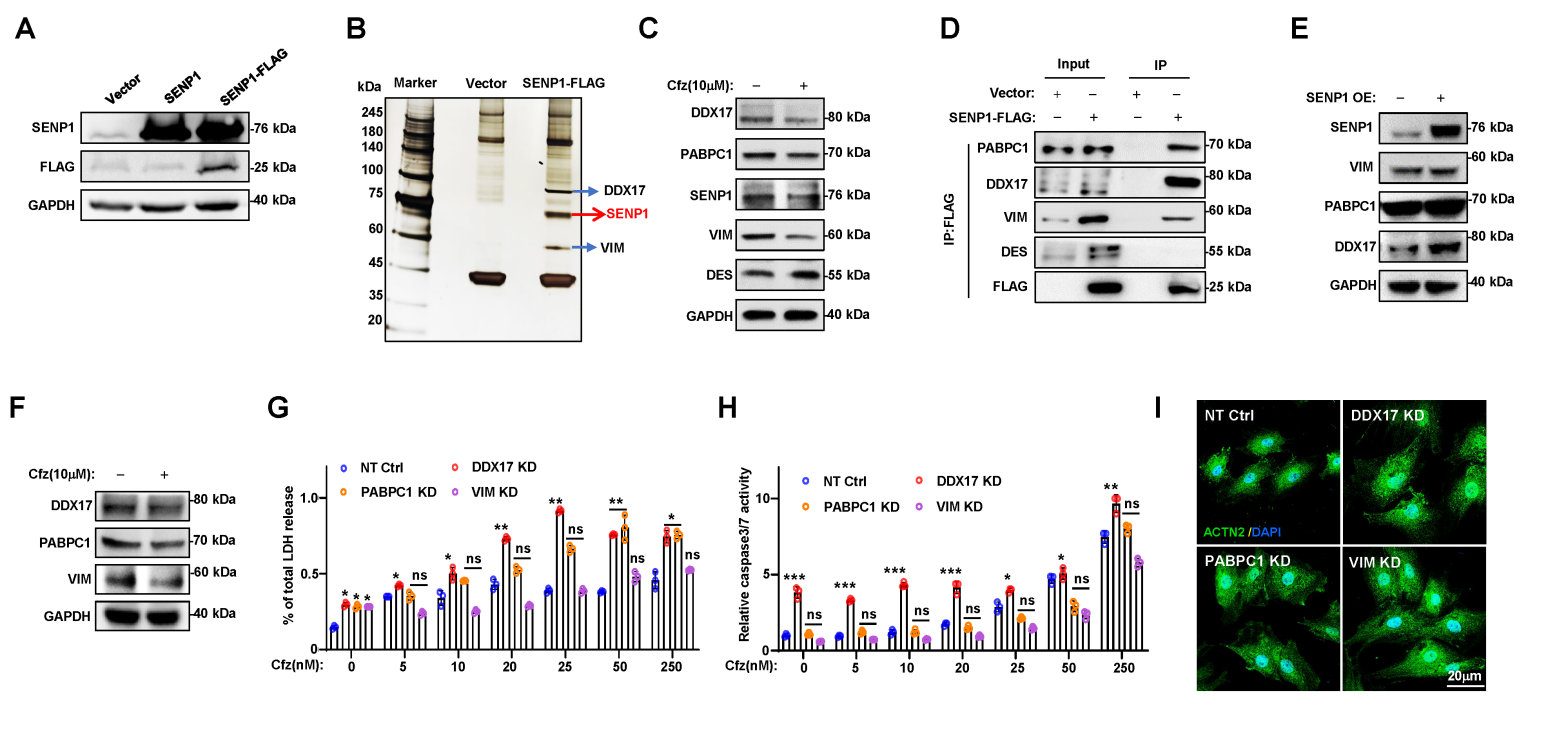
Figure 5 The interaction of SENP1 and DDX17 is involved in myocardial injury (A) Western blot analysis was used to validate the levels of SENP1 expression in NRVM cells transfected with vector or SENP1-flag overexpression plasmids. (B) Silver staining for Flag pull-down in vehicle- and Cfz-treated NRVM cells infected with lentivirus carrying SENP1-flags for 72 h. The red arrow indicates the SENP1 protein. The blue arrows indicate proteins identified through mass spectrometry (DDX17, 80 kDa; VIM, 60 kDa). (C) Representative western blots of DDX17, PABPC1, SENP1, VIM, and DES expressions in NRVM cells treated with 10 μM Cfz for 24 h (n = 3, biologically independent experiments). (D) Co-IP assay for Flag-SENP1 in H9c2 cells with anti-DDX17, anti-PABPC1, anti-VIM, and anti-DES antibodies. (E) Representative western blots of DDX17, PABPC1, and VIM expressions in H9c2 cells overexpressing SENP1. (F) Representative western blots of DDX17, PABPC1, and VIM expressions in H9c2 cells treated with 10 μM Cfz or vehicle. (G) Quantification of the percentage of total LDH release in NT Ctrl with DDX17 KD, PABPC1 KD, or VIM KD H9c2 cells treated with Cfz for 24 h by LDH cytotoxicity assay (n = 3). (H) Relative caspase3/7 activity in NT Ctrl with DDX17 KD, PABPC1 KD, or VIM KD H9c2 cells treated with Cfz for 24 h (n = 3). (I) Representative immunofluorescence confocal micrographs of the morphology of H9c2 cells (scale bar, 20 μm) transfected with DDX17 shRNA, PABPC1 shRNA, VIM shRNA or the negative control, as shown by ACTN2 (green) and DAPI, which were treated with vehicle or 10 μM Cfz for 24 h. Data are presented as the mean ± SEM and were analyzed by two-way ANOVA (G,H). *P < 0.05, **P < 0.01, ***P < 0.001.

To investigate the molecular mechanisms driving cardiac dysfunction caused by SENP1 deficiency, we analyzed SENP1 substrates in cardiomyocytes. We overexpressed a flag-tagged SENP1 fusion protein ( Figure 5A) and immunoprecipitated the complex in NRVM cells, followed by mass spectrometry-based proteomic analysis to identify the components of the interactome ( Figure 5B). We identified four partners, DEAD-box helicase 17 (DDX17), poly (A)-binding protein cytoplasmic 1 (PABPC1), vimentin (VIM), and desmin (DES); the first three were downregulated to various degrees by Cfz; however, DES was upregulated by Cfz treatment ( Figure 5C and Supplementary Table S1). To further validate the interaction between SENP1 and its partners, we performed a Co-IP assay in H9c2 cells and found that DDX17, PABPC1, and VIM, but not DES, interacted with SENP1 ( Figure 5D). Importantly, when SENP1 was overexpressed, DDK17, but not VIM or PABPC1, increased appreciably ( Figure 5E and Supplementary Figure S5A), whereas the DDK17 protein was most notably suppressed by Cfz treatment compared with VIM and PABPC1 ( Figure 5F and Supplementary Figure 5B), indicating that DDK17 is the most important effective substrate of SENP1. Next, we depleted VIM, PABPC1, and DDX17 expressions in H9c2 cells ( Supplementary Figure 5C–F) and confirmed that DDX17 deficiency had the most significant effect on the release of LDH, cleavage of Caspase3/7 activity, and morphological abnormalities after Cfz treatment ( Figure 5G–I). These results demonstrated that SENP1 participates in Cfz-induced cardiotoxicity by interacting with DDX17.

### SENP1 deSUMOylates DDX17 to reduce polyubiquitination

**Figure 6:**
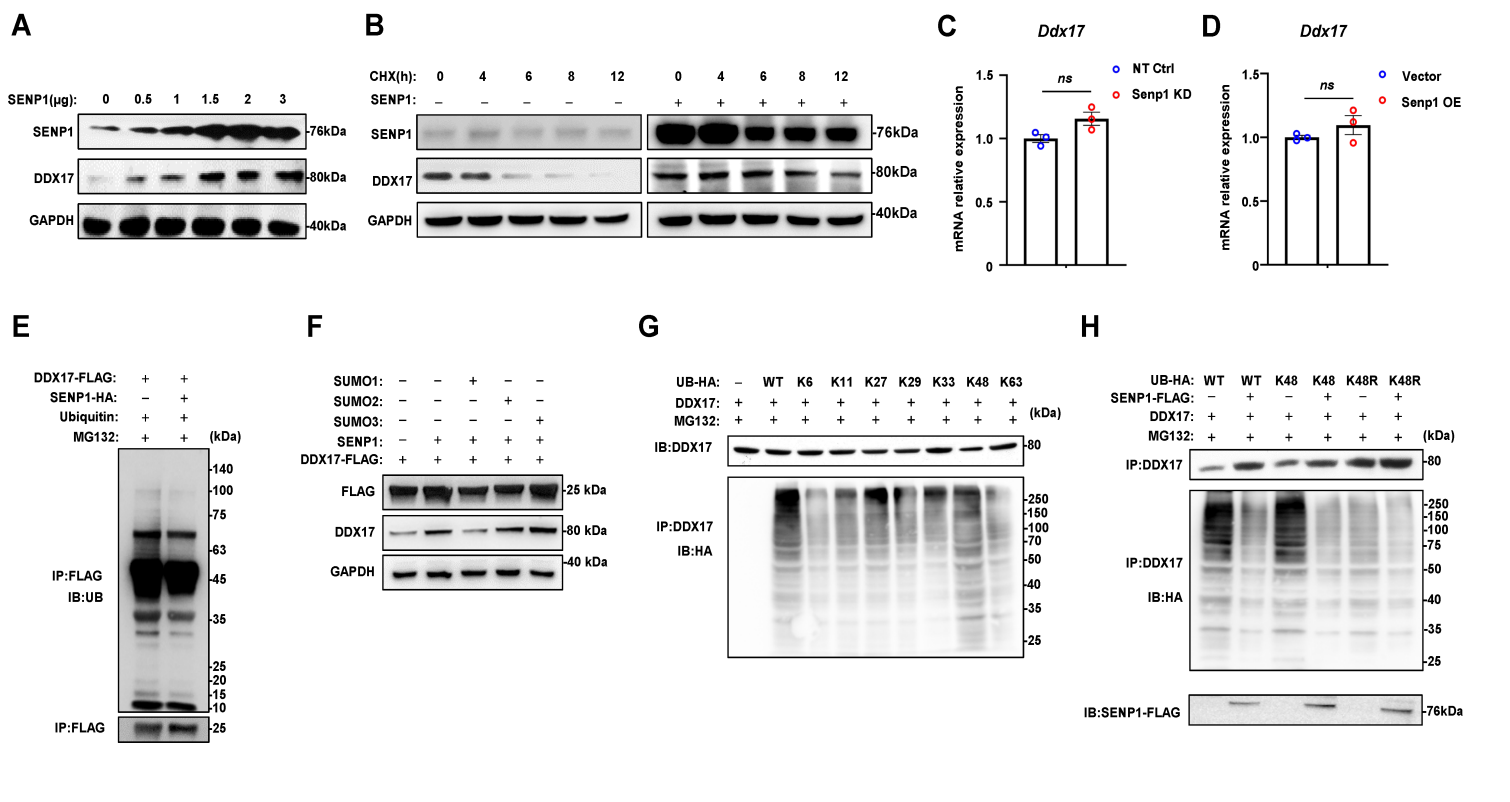
Figure 6 SENP1 alleviates SUMOylation and K48-linked polyubiquitination of the DDX17 protein (A) Levels of the DDX17 protein in H9c2 cells overexpressing SENP1. (B) Degradation rate of the DDX17 protein in the presence of SENP1 overexpression in H9c2 cells treated with 20 μM cycloheximide (CHX) for up to 12 h (n = 3 biologically independent experiments). (C,D) The mRNA level of Ddx17 was analyzed by RT-qPCR when knockdown or overexpressing of Senp1 in H9c2 cells. (E) Ubiquitination status of DDX17-FLAG in H9c2 cells transfected with the SENP1-HA overexpression plasmid or vehicle vector. (F) Representative western blots of DDX17 or flag expression in H9c2 cells transfected with the DDX17-FLAG, SENP1, SUMO1, SUMO2, or SUMO3-overexpression plasmid or vehicle vector. (G) Polyubiquitination linkage features of DDX17 mapped by positive ubiquitin mutations in H9c2 cells transfected with SENP1-FLAG overexpression plasmids. (H) K48-linked or K48R-linked polyubiquitination of DDX17 in H9c2 cells transfected with the SENP1-FLAG overexpression plasmid or vehicle vector. Data are presented as the mean ± SEM and were analyzed by Student’s t test (C,D). ns, not significant.

To understand how SENP1 regulates DDX17 protein levels, we overexpressed SENP1 in H9c2 cells in a dose-dependent manner and observed an increase in the DDX17 protein level ( Figure 6A and Supplementary Figure S6A), and the half-life of the DDX17 protein was significantly prolonged ( Figure 6B and Supplementary Figure S6B) without altering the transcriptional level of *DDX17* mRNA ( Figure 6C,D). SENP1 affects the proteasomal degradation of DDX17; thus, we detected the ubiquitination level of DDX17 and found that the overexpression of SENP1 indeed suppressed the ubiquitination of the DDX17 protein by removing SUMO1-mediated SUMOylation ( Figure 6E,F).

Next, we identified the amino acid residues responsible for DDX17 ubiquitination. Our results demonstrated that DDX17 degradation, including K11-, K27-, and K48-linked polyubiquitination, was linked to different types of ubiquitin-linked ubiquitination to some extent; however, the K48-linked peptide was dominant ( Figure 6G), which was also evidenced by different ubiquitination levels of DDX17 under positive or negative mutation of K48 on ubiquitin ( Figure 6H). These data suggest that SENP1 deSUMOylates DDX 17 protein stability by suppressing K48-linked polyubiquitination.

### Rescue of Senp1 partially ameliorates Cfz-induced myocardial injury

**Figure 7:**
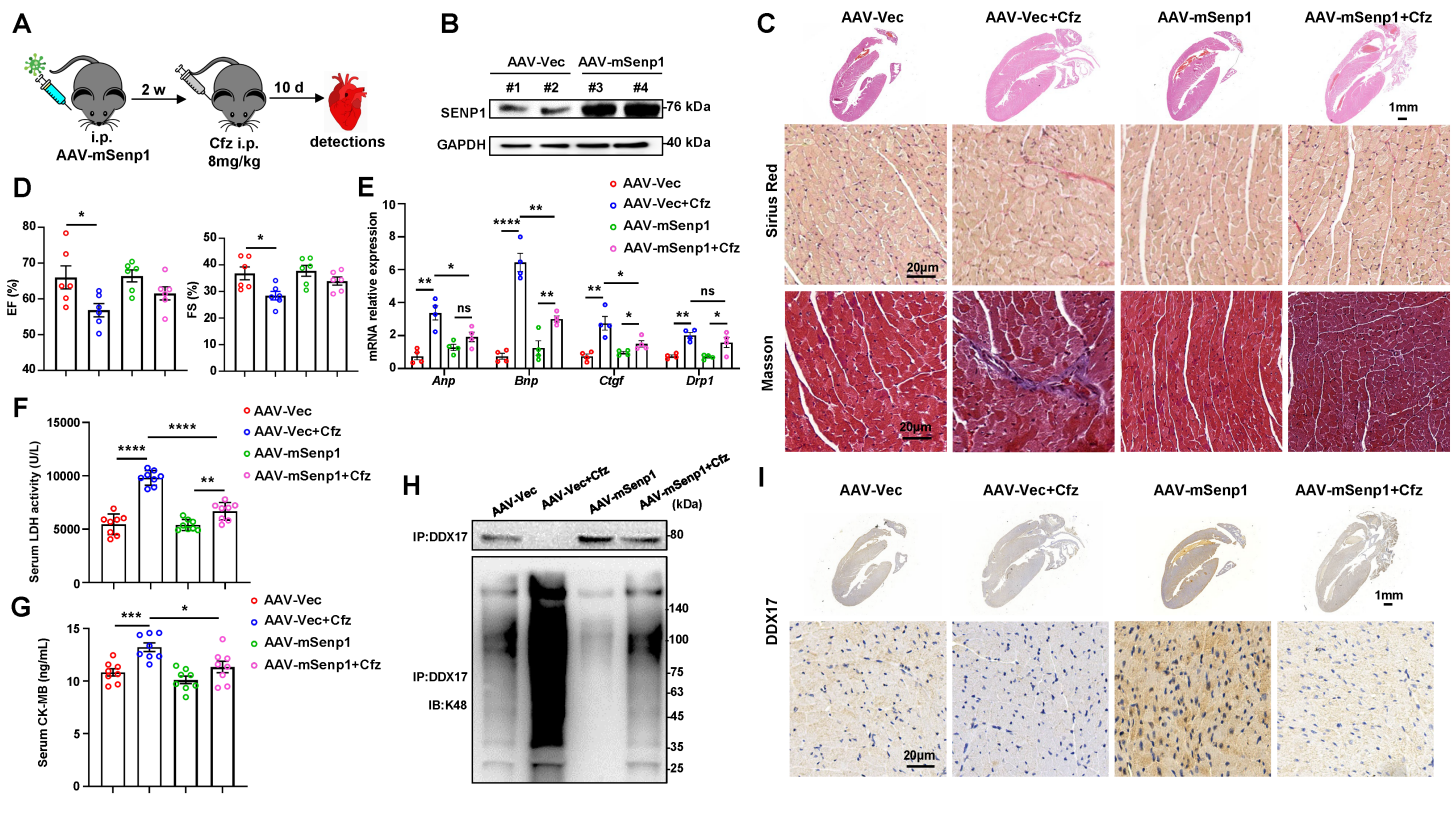
Figure 7 Rescue of Senp1 expression alleviates Cfz-induced cardiotoxicity (A) Experimental design and treatment schedule for AAV mSenp1 gene delivery in C57BL/6 mice followed by Cfz treatment (AAV-Vec group: n = 6; AAV-Vec + Cfz group: n = 6; AAV-mSenp1 group: n = 6; AAV-mSenp1 + Cfz group: n = 6). (B) Representative western blots of SENP1 protein levels after transduction with AAV mSenp1 or its blank vector for 2 weeks. (C) Representative images of H&E-stained heart longitudinal sections from mice transduced with AAV-Vec or AAV-mSenp1, which were treated with or without Cfz; scale bar, 1 mm. Representative histological sections from the hearts were stained with MTT and Sirius Red (scale bar, 20μm). (D) Echocardiographic assessment of EF and FS in AAV-transduced mice at 10 days after Cfz treatment (AAV-Vec group: n = 6; AAV-Vec + Cfz group: n = 6; AAV-mSenp1 group: n = 6; AAV-mSenp1 + Cfz group: n = 6). (E) The mRNA levels of Anp, Bnp, Ctgf and Drp1 in the left ventricles of the four groups were analyzed by RT-qPCR (n = 4/group). (F,G) Mouse serum LDH and CK-MB levels in the four groups (n = 8/group). (H) K48-linked polyubiquitination of DDX17 in the left ventricle of mouse hearts transduced with AAV-Vec or AAV-mSenp1, followed by treatment with vehicle or Cfz. (I) Representative immunohistochemical staining of DDX17 protein in heart tissues of AAV-transduced mice treated with vehicle or Cfz. Scale bar: 1 mm or 20 μm. Data are presented as the mean ± SEM and were analyzed by two-way ANOVA (E) and one-way ANOVA (D,F and G). *P < 0.05, **P < 0.01, ***P < 0.001, ****P < 0.0001. ns, not significant.

Considering the protective role of SENP1 in cardiomyocytes, we evaluated the potential of using a cardiac-specific AAV9 virus to restore Senp1 expression in mice to examine the ability of SENP1 to mitigate Cfz-induced cardiotoxicity ( Figure 7A). Two weeks after injection, we extracted tissue from mouse hearts and detected the protein levels of SENP1. Our data revealed that SENP1 was successfully overexpressed in heart tissues ( Figure 7B), indicating that the AAV virus significantly rescued the expression of SENP1. We investigated the effect of AAV-mSenp1 on reducing Cfz-induced cardiotoxicity in mice. Our findings revealed that the overexpression of SENP1 protected against Cfz-induced toxicity by decreasing cardiac fibrosis and myocardial injury while also restoring heart size and the arrangement of heart fibers ( Figure 7C). In addition, left ventricular functions, including EF and FS, were significantly improved in the AAV-mSenp1 group, and the expressions of the hypertrophic and fibrotic marker genes *Anp*, *Bnp* and *Ctgf* were also significantly lower than those of other downstream effectors of DDX17, such as *Drp1* ( Figure 7D,E), mainly because of the marked decrease in LDH release and CK-MB activity ( Figure 7F,G). Finally, the DDX17 protein, a substrate of SENP1, was also extensively upregulated following Cfz treatment, accompanied by a decrease in K48-linked polyubiquitination ( Figure 7H,I). Taken together, these results indicate that rescuing SENP1 expression in heart tissue can alleviate the cardiotoxicity of Cfz.

## Discussion

The cardiotoxicity of Cfz has long been recognized in the clinical management of patients with multiple myeloma; however, the underlying mechanisms have not yet been elucidated. With the development of a new interdiscipline, cardio-oncology, the toxicity of MM tumors and their treatment of the heart has received increasing attention in both clinical and basic research. Previous studies have shown that MM patients are at increased risk of serious cardiovascular adverse events and have a higher prevalence of serious cardiotoxicity when treated with PIs [ 3, 11] . In the present study, we confirmed the cardiotoxicity of Cfz through *in vivo* experiments in mice and cultured cardiomyocytes *in vitro*. We identified the suppressed expression of SENP1, a key enzyme involved in SUMOylation, and revealed a new mechanism by which SENP1 protects cardiomyocytes from PI-induced apoptosis from the perspective of posttranslational regulation of DDX17.

Previous studies have revealed the protective role of SENP1 in the heart by reducing oxidative stress [12], regulating mitochondrial function [13], and reprogramming cardiac remodeling [14]. However, the correlation between SENP1 and Cfz-induced cardiotoxicity has not been investigated. Our results revealed that Cfz triggered severe damage to cardiomyocytes when SENP1 was depleted, indicating that SENP1 is required for cardiomyocyte homeostasis. Another recent study by our group revealed that SENP1 inhibits the adverse ventricular remodeling response by inhibiting HSP90ab1 SUMOylation and stabilizing STAT3 [9]. In addition, activation of the Senp1/Sirt3 signaling pathway attenuates autophagy and cell death in diabetic cardiomyopathy [15], and SENP1 also protects against myocardial ischemia/reperfusion injury via a HIF1α-dependent pathway [16]. Together with our findings, these studies illustrate the important role of SENP1 in maintaining cardiomyocyte function.

DDX17, a member of a large family of DEAD-box RNA helicases, is essential for RNA metabolism, including mRNA splicing, export, and transcription [ 17, 18] . An updated study demonstrated that DDX17 protects against heart failure by maintaining mitochondrial homeostasis via the BCL6-DRP1 pathway [19]. DDX17 also eliminates ROS production and ameliorates cardiomyocyte apoptosis by promoting PI3K/Akt phosphorylation [20]. Additionally, deSUMOylation of DDX17 increases its stability, promotes its interaction with partner proteins, and enhances DDX17-mediated transcriptional repression [21]. Interestingly, we identified a promising candidate, DDX17, which interacts with and can be modified by SENP1 in cardiomyocytes. These findings suggest that SENP1 may regulate the activity of DDX17 through direct interaction and removal of SUMO modifications. To the best of our knowledge, this is the first report on the machinery of the SENP1-DDX17 axis in regulating Cfz-induced cardiotoxicity.

Nevertheless, our study has several limitations, which are reflected in the following aspects: we investigated neither the reason why Senp1 expression is suppressed by Cfz nor the roles of other SENP family molecules in Cfz-induced myocardial injury, as reports suggest that SENP5 also has similar cardioprotective effects [22], but we did not observe any significant changes in its levels. Second, we did not validate our findings in clinical patients or large sample cohorts, including the expression levels of SENP1 and their relationship with patient survival time and treatment prognosis. Third, we did not test whether other PIs, such as bortezomib and ixazomib, exert a mechanism similar to that of Cfz. These aspects require further investigation by clinical and basic researchers.

In summary, our study revealed a crucial protective role of the SENP1-DDX17 axis in Cfz-induced cardiotoxicity, providing a new perspective for understanding the molecular mechanism of proteasome inhibitor-induced cardiotoxicity. Our study also highlights that developing a cardiomyocyte-targeted gene intervention strategy may be an effective and novel approach to reverse Cfz-triggered myocardial fiber remodeling and attenuate the progression of cardiac injury.

## Supporting information

25410Revised_supplementary_figures

25410Supplementary_Resources

25410Supplementary_Table

## Supplementary Data

Supplementary data are available at *Acta Biochimica et Biophysica Sinica* online.
