# Supplementary material for: Carfilzomib triggers cardiotoxicity by suppressing SENP1-mediated deSUMOylation of DDX17: SENP1 alleviates Cfz cardiotoxicity
Source: Acta Biochim Biophys Sin (Shanghai). 2025 Oct 9;58(7):1499–508. doi: 10.3724/abbs.2025121 (PMC13449089; doi:10.3724/abbs.2025121)
Supplement: Supplementary file 1 [file 25410Revised_supplementary_figures.docx]

**Supplementary Figure S1. Changes in heart, liver and kidney functions of a Cfz-induced cardiotoxicity model in mice** (A) Echocardiographic assessment of left ventricular systolic posterior wall thickness (LVPWs), (B) left ventricular systolic internal dimensions (LVIDs), (C) interventricular septal thickness at systole (IVSs), (D) left ventricular diastolic posterior wall thickness (LVPWd), (E) left ventricular diastolic internal dimensions (LVIDd), (F) interventricular septal thickness at diastole (IVSd) in mice (Cfz group: *n* = 14; vehicle group: *n* = 10). Serum ALT (G), ALB (H), BUN (I) and Cr (J) levels were measured by ELISA in each group. (K) Representative images of H&E-stained liver longitudinal sections from vehicle- and Cfz-treated mice (*n* = 4); scale bar, 500μm and 20μm. (L) Representative images of H&E-stained kidney longitudinal sections from vehicle- and Cfz-treated mice (*n* = 4); scale bar, 1 mm and 20 μm. Data are presented as the mean±SEM and were analyzed by two-side Student’s *t* test. ***P*＜0.01, ****P*＜0.001.

**Supplementary Figure S2. Isolation and identification of primary rat cardiomyocytes and changes in cell viability after Cfz treatment** (A) Isolation and differentiation of left ventricular myocytes from neonatal rat hearts and identification of cardiac fibroblasts by H&E staining and immunofluorescence (IF) for myocardial cell markers ACTN2, cTnT, Vimentin (all in green); scale bar: 20 μm. (B) The cell viability of H9c2 or NRVM cells with Cfz treatment by CCK-8 assay (*n* = 3). Data are presented as the mean ± SEM

**Supplementary Figure S3. RNA-Sequencing related bioinformatics analysis in NRVMs** (A) Gene Ontology (GO) analysis of top 20 enriched terms of the differential expressed genes (DEGs) about cellular component. (B) GO analysis of top 20 enriched terms of the DEGs about molecular function. (C) GO analysis of top 20 enriched terms of the DEGs about biological process. (D) GSEA analysis shows genes enrichment in proteasome, SUMO transferase activity and ubiquitin-protein transferase activity in Cfz-treated NRVM cells.

**Supplementary Figure S4. Cfz treatment causes no changes in other members of the SENP family** (A) The mRNA levels of *Senp2*, *Senp3*, *Senp6* and *Senp7* in the vehicle- and Cfz-treated group were analyzed by RT-qPCR (*n* = 8). (B) Representative western blots of SENP5 protein levels and quantification results in the vehicle- and Cfz-treated group (*n* = 5). Data are presented as the mean ± SEM and were analyzed by two-way ANOVA (A) and two-side Student’s *t* test (B).

**Supplementary Figure S5. Verification of the expressions of DDX17, PABPC1 and VIM in H9c2 cells** (A,B) The quantification results of **Figure 5E** (A) and **Figure 5F** (B). (C−E) Representative western blot images in H9c2 cells with *VIM* (C), *PABPC1* (D) and *DDX17* (E) knockdown. **(**F**)** RT-qPCR validating levels of *Ddx17*, *Pabpc1* or *Vim* expressions in H9c2 cells. Data are presented as the mean ± SEM and were analyzed by two-way ANOVA (A, B and F) (*n* = 3 biologically independent experiments).

**Supplementary Figure S6. SENP1 promotes the stability of DDX17** (A,B) The quantification results of **Figure 6A** (A) and **Figure 6B** (B). Data are presented as the mean ± SEM and were analyzed by two-way ANOVA (*n* = 3 biologically independent experiments).
