# Supplementary material for: Carfilzomib triggers cardiotoxicity by suppressing SENP1-mediated deSUMOylation of DDX17: SENP1 alleviates Cfz cardiotoxicity
Source: Acta Biochim Biophys Sin (Shanghai). 2025 Oct 9;58(7):1499–508. doi: 10.3724/abbs.2025121 (PMC13449089; doi:10.3724/abbs.2025121)
Supplement: Supplementary file 2 [file 25410Supplementary_Resources.doc]

**Key resources of this study**

| **Category** | **Source** | **Cat. No.** |
| --- | --- | --- |
| **Antibodies** | | |
| ACTN2 Rabbit mAb | ABclonal Technology | A8939 |
| Cardiac troponin T (TNNT2) Rabbit mAb | ABclonal Technology | A4914 |
| Vimentin Rabbit pAb | ABclonal Technology | A21648 |
| GAPDH (14C10) Rabbit mAb | Cell signaling technology | #2118 |
| DDX17 (H-7) Antibody | Santa Cruz Biotechnology | sc-398168 |
| DDX17 Rabbit mAb | ABclonal Technology | A5731 |
| DDX17 Rabbit pAb | ABclonal Technology | A17078 |
| SENP1 (C-12) Antibody | Santa Cruz Biotechnology | sc-271360 |
| SENP1 Polyclonal Antibody | Immunoway | YT4236 |
| SENP5-Specific Polyclonal antibody | Proteintech | 19529-1-AP |
| pAbPC1 Rabbit pAb | ABclonal Technology | A14872 |
| Desmin Rabbit mAb | ABclonal Technology | A3736 |
| Ubiquitin (E6K4Y) XP® Rabbit mAb | Cell Signaling Technology | #20326 |
| K48-linkage Specific Polyubiquitin (D9D5) Rabbit mAb | Cell Signaling Technology | #8081 |
| Mouse anti HA-Tag mAb | ABclonal Technology | AE008 |
| DYKDDDDK Tag (D6W5B) Rabbit mAb | Cell signaling technology | #14793 |
| SUMO-1 Antibody | Cell signaling technology | #4930 |
| SUMO-2/3 (18H8) Rabbit mAb | Cell signaling technology | #4971 |
| Alexa Fluor® 488 donkey anti-Rabbit IgG (H + L) Antibody | Invitrogen | R37118 |
| [HRP-conjugated Goat anti-Rabbit IgG (H+L)](https://abclonal.com.cn/catalog/AS014) Antibody | ABclonal Technology | AS014 |
| [HRP-conjugated Goat anti-Mouse IgG (H+L)](https://abclonal.com.cn/catalog/AS014) Antibody | ABclonal Technology | AS003 |
| **Chemicals, peptides and recombinant proteins** | | |
| Recombinant SENP1 protein | Abcam | ab268953 |
| Protein A/G agarose beads | Thermo Fisher Scientific | 78609 |
| Carfilzomib | MedChemExpress | HY-10455 |
| ANTI-FLAG® M2 Affinity Gel | Sigma-Aldrich | A2220 |
| TRIzol Reagent | Invitrogen | 15596018CN |
| Cycloheximide | SelleckChem | S7418 |
| MG132 | SelleckChem | S2619 |
| UltraCruz^®^ Hard-set Mounting Medium with DAPI | Santa Cruz Biotechnology | sc-359850 |
| Corn oil | MedChemExpress | HY-Y1888 |
| DMSO | Sigma-Aldrich | D2650 |
| cOmplete™ protease inhibitor cocktail | Roche | [04693132001](https://www.sigmaaldrich.cn/CN/zh/product/roche/04693132001) |
| Pierce Protein G plus Agrose | Sigma-Aldrich | #22852 |
| Opti-MEM®I(1×) Reduced Serum | Gibco | 31985070 |
| Polyethylenimine(PEI) | Polysciences | 23699-1 |
| Hexadimethrine bromide(polybrene) | Sigma-Aldrich | 107689 |
| 1Kb DNA Ladder | Genstar | M014 |
| 100bp DNA ladder | Genstar | M016 |
| All-In-One 5X RT MasterMix | Abm | G592 |
| EvaGreen 2× qPCR MasterMix | Abm | Mastermix-LR |
| Neutral Buffered Formalin Fixative | Solarbio | G2161 |
| 4% paraformaldehyde | Solarbio | P1110 |
| 5-bromo-2-deoxyuridine | Sigma-Aldrich | B5002 |
| Poly(ethylene glycol)8000 | Sigma-Aldrich | 89510 |
| 3xFLAG peptide | Sigma-Aldrich | F4799 |
| FLAG Peptide | Sigma-Aldrich | F3290 |
| Protein A/G magnetic | YEASEN | 36417ES |
| SuperSignal™ West Pico PLUS chemiluminescence substrate | Thermo Fisher Scientific | 34580 |
| 2 × Phanta Max Master Mix | Vazyme Biotech | P515 |
| Optiprep Iodixanol solution | Sigma-Aldrich | D1556 |
| **Enzymes** | | |
| RNase A, DNase and protease-free | Thermo Fisher | EN0531 |
| Proteinase K Solution, ChIP grade | Thermo Fisher | 26160 |
| Benzonase Nuclease | Sigma-Aldrich | E1014-25KU |
| FastAP Thermosensitive Alkaline Phosphatase | Thermo Fisher Scientific | EF0651 |
| AgeI | NewEngland Biolabs | R0552S |
| NotI-HF | NewEngland Biolabs | R3189S |
| BamHI | NewEngland Biolabs | R0136S |
| XbaI | NewEngland Biolabs | R0145S |
| EcoRI | NewEngland Biolabs | R0101S |
| KpnI-HF | NewEngland Biolabs | R3142S |
| HindIII-HF | NewEngland Biolabs | R3104S |
| XhoI | NewEngland Biolabs | R0146S |
| NcoI | NewEngland Biolabs | R0193S |
| CutSmart Buffer | NewEngland Biolabs | 137204S |
| NEBuffer1 | NewEngland Biolabs | B7001S |
| NEBuffer2 | NewEngland Biolabs | B7002S |
| NEBuffer3 | NewEngland Biolabs | B7003S |
| NEBuffer4 | NewEngland Biolabs | B7004S |
| T4 DNA Ligase | NewEngland Biolabs | M0202S |
| 10×Buffer for T4 DNA ligase | NewEngland Biolabs | B0202S |
| **Plasmids** | | |
| pLKO.1 | Addgene | |
| Senp1 sh#1 | Self-construction | |
| Senp1 sh#2 | Self-construction | |
| Senp1 sh#3 | Self-construction | |
| Vim sh#1 | Self-construction | |
| Vim sh#2 | Self-construction | |
| Vim sh#3 | Self-construction | |
| Pabpc1 sh#1 | Self-construction | |
| Pabpc1 sh#2 | Self-construction | |
| Pabpc1 sh#3 | Self-construction | |
| Pabpc1 sh#4 | Self-construction | |
| Ddx17 sh#1 | Self-construction | |
| Ddx17 sh#2 | Self-construction | |
| Ddx17 sh#3 | Self-construction | |
| Ddx17 sh#4 | Self-construction | |
| pITA insert | Gift from Dr. Yupeng Chen, Tianjin Medical University | |
| pITA-hSENP1-HA | Addgene | |
| pITA-hSENP1-flag | Self-construction | |
| pITA-rSENP1-flag | Self-construction | |
| pITA-rSENP1 | Self-construction | |
| pITA-hDDX17-flag | Self-construction | |
| pITA-hDDX17 | Self-construction | |
| SUMO1-HA | Addgene | |
| SUMO2-HA | Addgene | |
| SUMO3-HA | Addgene | |
| Ub-WT-HA | Addgene | |
| Ub-K6-HA | Addgene | |
| Ub-K11-HA | Addgene | |
| Ub-K27-HA | Addgene | |
| Ub-K29-HA | Addgene | |
| Ub-K33-HA | Addgene | |
| Ub-K48-HA | Addgene | |
| Ub-K63-HA | Addgene | |
| Ub-K48R-HA | Addgene | |
| psPAX_2_ | Gift from Dr. Xudong Wu, Tianjin Medical University, Dept. Cell Biology | |
| pMD2.G | Gift from Dr. Xudong Wu, Tianjin Medical University, Dept. Cell Biology | |
| pAAV-m*Senp1* | Self-construction | |
| pAAV-m*Senp1*-flag | Self-construction | |
| pAAV-MCS2 | Addgene | |
| pHelper | Addgene | |
| pAAV2/9n | Addgene | |
| **Critical commercial assays** | | |
| Pierce™ Silver Stain Kit | Thermo fisher Scientific | 24612 |
| Cell Counting Kit-8 (CCK-8) | APExBIO Technology | K1018 |
| LDH Cytotoxicity Assay Kit | Beyotime | C0017 |
| Lactate Dehydrogenase (LDH) Assay Kit | Jiancheng Bio | A020-2 |
| Aspartate Aminotransferase (AST) Assay Kit | Jiancheng Bio | C010-2-1 |
| Mouse Creatine Kinase MB Isoenzyme (CK-MB) Elisa Kit | Jiancheng Bio | H197-1-2 |
| Alanine Aminotransferase (ALT) Assay Kit | Jiancheng Bio | C009-2-1 |
| Albumin (ALB) Assay Kit | Jiancheng Bio | A028-2-1 |
| Blood Urea Nitrogen (BUN) Assay Kit | Jiancheng Bio | C013-3-1 |
| Creatinine (Cr) Assay Kit | Jiancheng Bio | C011-2-1 |
| Hematoxylin and Eosin (H&E) Kit | Solarbio | G1100 |
| Masson’s Trichrome Kit | Solarbio | G1340 |
| Sirius red staining Kit | G-CLONE | RS1220 |
| Pierce BCA Protein Assay Kit | Thermo fisher Scientific | 23225 |
| DeadEnd™ Fluorometric TUNEL System | Promega | G3250 |
| Caspase-Glo®3/7 Assay | Promega | G8091 |
| SPARKeasySuperpureMiniPlasmidKit | SparkJade | AD0102-C |
| Endofree Maxi Plasmid Kit | TIANGEN Biotech | DP117 |
| EnVision G12 DoublestainSystem,Rabbit/Mouse(DAB+/Permanent Red) | Dako | K5361 |
| FastPure ® Gel DNAExtraction Mini Kit | VazymeBiotech | DC301 |
| **Primers sequence(5**′→**3**′**)** | | |
| Rat *Gapdh*-F | CCGCATCTTCTTGTGCAGTG | |
| Rat *Gapdh*-R | GAGAAGGCAGCCCTGGTAAC | |
| Rat *Anp*-F | ACCTGCTAGACCACCTGGAG | |
| Rat *Anp*-R | CCTTGGCTGTTATCTTCGGTACCGG | |
| Rat *Bnp*-F | GAGGTCACTCCTATCCTCTGG | |
| Rat *Bnp*-R | GCCATTTCCTCCGACTTTTCTC | |
| Rat *Senp1*-F | TGCAGTGCTTGATTCCGTAG | |
| Rat *Senp1*-R | TTCTTTGTCCAGCGTTTCAC | |
| Rat *Senp5*-F | ACTGTTTTAGGGTTCCTAGACGA | |
| Rat *Senp5*-R | CCAGGTCGTCCATATCCAGC | |
| Rat *Ctgf*-F | AGGCTGCGAGTACAAGCTTC | |
| Rat *Ctgf*-R | GAGGCGGTAGTAGGCGGATG | |
| Rat *Ddx17*-F | CGGCTGCTACTTCGACATCA | |
| Rat *Ddx17*-R | GAGCTGGAGGTGAGCTGAAG | |
| Rat *Pabpc1*-F | AGGCTGGCTCTGATGAAGAG | |
| Rat *Pabpc1*-R | TCTGCTGCTGCTGCTGCTGT | |
| Rat *Vim*-F | AGGCAATGCGTGAGAAGAGA | |
| Rat *Vim*-R | TCTGCTGCTGCTGCTGCTGT | |
| Mouse *Gapdh*-F | ATCATCCCTGCATCCACT | |
| Mouse *Gapdh*-R | ATCCACGACGGACACATT | |
| Mouse *Anp*-F | TCGTCTTGGCCTTTTGGCT | |
| Mouse *Anp*-R | TCCAGGTGGTCTAGCAGGTTCT | |
| Mouse *Bnp*-F | AAGTCCTAGCCAGTCTCCAGA | |
| Mouse *Bnp*-R | GAGCTGTCTCTGGGCCATTTC | |
| Mouse *Senp1*-F | AGTAAAGAAGGTTCCGGTTCCCG | |
| Mouse *Senp1*-R | GCCGCCACTCACCGAAC | |
| Mouse *Senp2*-F | GCTAAGGTTCTCGGCACCATT | |
| Mouse *Senp2*-R | ATTACAAGCAGAAGACACCATG | |
| Mouse *Senp3*-F | CCGGCCATCTTTTGATGCCT | |
| Mouse *Senp3*-R | GCGAGGTGCTTTTTGAGTAGAG | |
| Mouse *Senp5*-F | GACACATTCCCTGACCAGCA | |
| Mouse *Senp5*-R | TCATCGCTGACAGCACTTGT | |
| Mouse *Senp6*-F | AGCGGCTCAGAGCAATCAG | |
| Mouse *Senp6*-R | AGGGCTGTTTTCAGCTTTGTG | |
| Mouse *Senp7*-F | CGGCGAGCTTCATCAGAGATT | |
| Mouse *Senp7*-R | TGGACAATGGTGACTGAGCAA | |
| Mouse *Drp1*-F | ATGCCAGCAAGTCCACAGAA | |
| Mouse *Drp1*-R | TGTTCTCGGGCAGACAGTTT | |
| Mouse *Ctgf*-F | TGACCTGGAGGAAAACATTAAGA | |
| Mouse *Ctgf*-R | AGCCCTGTATGTCTTCACACTG | |
| Mouse *Ddx17*-F | GGAGGCCGCTCACGATAC | |
| Mouse *Ddx17*-R | GGTGCCTTGAGCATAGGTGT | |
| Rat *Vim*-sh#1-F | CCGGGCTAACTACCAGGACACTATTCTCGAGAATAGTGTCCTGGTAGTTAGCTTTTTG | |
| Rat *Vim*-sh#1-R | AATTCAAAAAGCTAACTACCAGGACACTATTCTCGAGAATAGTGTCCTGGTAGTTAGC | |
| Rat *Vim*-sh#2-F | CCGGGGAGTCACTTCCTCTGGTTGACTCGAGTCAACCAGAGGAAGTGACTCCTTTTTG | |
| Rat *Vim*-sh#2-R | AATTCAAAAAGGAGTCACTTCCTCTGGTTGACTCGAGTCAACCAGAGGAAGTGACTCC | |
| Rat *Vim*-sh#3-F | CCGGGCGAAGTGGATGCCCTTAAAGCTCGAGCTTTAAGGGCATCCACTTCGCTTTTTG | |
| Rat *Vim*-sh#3-R | AATTCAAAAAGCGAAGTGGATGCCCTTAAAGCTCGAGCTTTAAGGGCATCCACTTCGC | |
| Rat *Ddx17*-sh#1-F | CCGGGGTCTTTGCCTTCCATCATGCCTCGAGGCATGATGGAAGGCAAAGACCTTTTTG | |
| Rat *Ddx17*-sh#1-R | AATTCAAAAAGGTCTTTGCCTTCCATCATGCCTCGAGGCATGATGGAAGGCAAAGACC | |
| Rat *Ddx17*-sh#2-F | CCGGGCTGATGGATCAGCACTTTACCTCGAGGTAAAGTGCTGATCCATCAGCTTTTTG | |
| Rat *Ddx17*-sh#2-R | AATTCAAAAAGCTGATGGATCAGCACTTTACCTCGAGGTAAAGTGCTGATCCATCAGC | |
| Rat *Ddx17*-sh#3-F | CCGGGGATCAGCACTTTACAGAACCCTCGAGGGTTCTGTAAAGTGCTGATCCTTTTTG | |
| Rat *Ddx17*-sh#3-R | AATTCAAAAAGGATCAGCACTTTACAGAACCCTCGAGGGTTCTGTAAAGTGCTGATCC | |
| Rat *Ddx17*-sh#4-F | CCGGGCAGGGATATGGTTGGCATTGCTCGAGCAATGCCAACCATATCCCTGCTTTTTG | |
| Rat *Ddx17*-sh#4-R | AATTCAAAAAGCAGGGATATGGTTGGCATTGCTCGAGCAATGCCAACCATATCCCTGC | |
| Rat *Pabpc1*-sh#1-F | CCGGGCAAGCCAGTACGCATCATGTCTCGAGACATGATGCGTACTGGCTTGCTTTTTG | |
| Rat *Pabpc1*-sh#1-R | AATTCAAAAAGCAAGCCAGTACGCATCATGTCTCGAGACATGATGCGTACTGGCTTGC | |
| Rat *Pabpc1*-sh#2-F | CCGGGCTCCAAGGGCTATGGATTTGCTCGAGCAAATCCATAGCCCTTGGAGCTTTTTG | |
| Rat *Pabpc1*-sh#2-R | AATTCAAAAAGCTCCAAGGGCTATGGATTTGCTCGAGCAAATCCATAGCCCTTGGAGC | |
| Rat *Pabpc1*-sh#3-F | CCGGGCAAGTTTGGGCCTGCCTTAACTCGAGTTAAGGCAGGCCCAAACTTGCTTTTTG | |
| Rat *Pabpc1*-sh#3-R | AATTCAAAAAGCAAGTTTGGGCCTGCCTTAACTCGAGTTAAGGCAGGCCCAAACTTGC | |
| Rat *Pabpc1*-sh#4-F | CCGGGCATGAAGATGCGCAGAAAGCCTCGAGGCTTTCTGCGCATCTTCATGCTTTTTG | |
| Rat *Pabpc1*-sh#4-R | AATTCAAAAAGCATGAAGATGCGCAGAAAGCCTCGAGGCTTTCTGCGCATCTTCATGC | |
| Rat *Senp1*-sh#1-F | CCGGGATACCTCAGTCCATTGAAGACTCGAGTCTTCAATGGACTGAGGTATCTTTTTG | |
| Rat *Senp1*-sh#1-R | AATTCAAAAAGATACCTCAGTCCATTGAAGACTCGAGTCTTCAATGGACTGAGGTATC | |
| Rat *Senp1*-sh#2-F | CCGGACCTCAGTCCATTGAAGAGAACTCGAGTTCTCTTCAATGGACTGAGGTTTTTTG | |
| Rat *Senp1*-sh#2-R | AATTCAAAAAACCTCAGTCCATTGAAGAGAACTCGAGTTCTCTTCAATGGACTGAGGT | |
| Rat *Senp1*-sh#3-F | CCGGGGATGGAGAGAGCATTCTTCACTCGAG TGAAGAATGCTCTCTCCATCCTTTTTG | |
| Rat *Senp1*-sh#3-R | AATTCAAAAAGGATGGAGAGAGCATTCTTCACTCGAG TGAAGAATGCTCTCTCCATCC | |
| pITA-h*SENP1*-flag-XbaI-F | GCTCTAGAATGGATTACAAGGACGACGATGACAAGGATGATATTGCTGATAG | |
| pITA-h*SENP1*-KpnI-R | CGGGTACCTCACAAGAGTTTTCGGTGGA | |
| pITA-r*Senp1*-NotI-F | AAATATGCGGCCGCATGGAGGACACTGCCGAC | |
| pITA-r*Senp1*-flag-NotI-F | AAATATGCGGCCGCATGGATTACAAGGACGACGATGACAAGGAGGACACTGCCGACGGG | |
| pITA-r*Senp1*-XbaI-R | CGTCTAGATCACAAGAGCTTCCGGTGGA | |
| pITA-h*DDX17*-NotI-F | AAATATGCGGCCGCATGCCCACCGGCTTTGTA | |
| pITA-h*DDX17*-flag-NotI-F | AAATATGCGGCCGCATGGATTACAAGGACGACGATGACAAGCCCACCGGCTTTGTAGCC | |
| pITA-h*DDX17*-BamHI-R | CGGGATCCTCATTTACGTGAAGGAGGAG | |
| pAAV-m*Senp1*-flag-EcoRI-F | GCGAATTCATGGATTACAAGGACGACGATGACAAGAAAACCCCCTTAACTGCCGCC | |
| pAAV-m*Senp1*-EcoRI-F | GCGAATTCATGAAAACCCCCTTAACT | |
| pAAV-m*Senp1*-NcoI-R | CGCCATGGTCACAAGAGCTTCCGGTGGA | |
| AAV2 ITR-F | GGAACCCCTAGTGATGGAGTT | |
| AAV2 ITR-R | CGGCCTCAGTGAGCGA | |
